# Nurses' and Probationers' Food

**Published:** 1886-10-30

**Authors:** Louisa Twining


### NURSES' AND PROBATIONERS' FOOD.

I WAS glad to see, in the first number of THE HOSPITAL, a discussion was opened on the subject of diet of nurses and officers (as well as patients), and I thought it must be forgotten that a paper was read only last year, and the matter discussed. I therefore send you a copy of it from the Journal of the Association, as it hardly seems necessary to have to repeat what I then said. I have given away all the separate copies which I had printed at the time. *Much* experience since has confirmed all I stated, and indeed much more might be added—especially as to the terrible under-feeding in homes for probationers, causing waste of health and strength.

I consider the dietary given in the first number very deficient in puddings, and fruits and vegetables, all of which are preferred by nurses to so much meat, which *many* turn against, and there is thus great waste.

LOUISA TWINING.

